# Supplementary material for: How about an Educational Framework for Nursing Staff in Long-Term Care Facilities to Improve the Care of Behavioral and Psychological Symptoms of Dementia?
Source: Int J Environ Res Public Health. 2022 Aug 23;19(17):10493. doi: 10.3390/ijerph191710493 (PMC9518535; doi:10.3390/ijerph191710493)
Supplement: Supplementary file 1 [file ijerph-19-10493-s001.zip › ijerph-1864558-supplementary.pdf]

COREQ checklist

Title: How about an Educational Framework for Nursing Staff in Long-Term Care Facilities to improve the Care of Behavioral and Psychological Symptoms of Dementia?

Developed from: Tong, A., Sainsbury, P., & Craig, J. (2007). Consolidated criteria for reporting qualitative research (COREQ): a 32-item checklist for interviews and focus groups [34].

No. Item	Guide questions / description	Reported on page # and remark
**Domain 1: Research team and reflexivity**		
Personal Characteristics		
1. Interviewer/facilitator	Which author/s conducted the interview or focus group?	Page 12 (Author Contributions on Investigation)
2. Credentials	What were the researcher's credentials? (E.g., PhD, MD)	D.K., MSN; Y.-R.C., PhD; Y.-N.L., PhD; W.-H.P., PhD; S.-O.C., PhD
3. Occupation	What was their occupation at the time of the study?	D.K., PhD student, Y.-R.C., research professor, Y.-N.L., associate professor, W.-H.P., lecturer, and S.-O.C., professor
4. Gender	Was the researcher male or female?	Female
5. Experience and training	What experience or training did the researcher have?	All researchers have sufficient experience in participating in training programs about qualitative research.<br>The corresponding author has taught qualitative research methods to graduate students for more than 10 years.
Relationship established		
6. Relationship established	Was a relationship established prior to study commencement?	No
7. Participant knowledge of the interviewer	What did the participants know about the researcher? E.g., personal goals, reasons for doing the research	Page 6 (Ethical Considerations)
8. Interviewer characteristics	What characteristics were reported about the interviewer/facilitator? E.g., bias, assumptions, reasons, and interests in the research topic	Page 5
**Domain 2: Study design**		
Theoretical framework		
9. Methodological orientation and theory	What methodological orientation was stated to underpin the study? E.g., grounded study, discourse analysis, ethnography, phenomenology, content analysis	Page 5 (directed content analysis)
Participant selection		
10. Sampling	How were participants selected? E.g., purposive, convenience, consecutive, snowball	Page 4 (purposive sampling)

11. Method of approach	How were participants approached? E.g., face-to-face, telephone, mail, email	Page 5
12. Sample size	How many participants were in the study?	Page 5 and Table 1
13. Non-participation	How many people refused to participate or dropped out? Reasons?	None
Setting		
14. Setting of data collection	Where was the data collected? E.g., home, clinic, workplace	Depends on the participants (all data were collected remotely, so the place was possible anywhere as long as it wasn't so noisy.)
15. Presence of non-participants	Was anyone else present besides the participants and researchers?	None
16. Description of sample	What are the important characteristics of the sample? E.g., demographic data, date	Page 4 and Table 1
Data collection		
17. Interview guide	Were questions, prompts, guides provided by the authors? Was it pilot tested?	Interview questions were provided on Page 5.<br>No pilot tested.
18. Repeat interviews	Were repeat interviews carried out? If yes, how many?	No repeated interviews.
19. Audio/visual recording	Did the research use audio or visual recording to collect the data?	Page 5 (Audio)
20. Field notes	Were field notes made during and/or after the interview?	No
21. Duration	What was the duration of the interviews or focus group?	Page 5 (60 to 90 minutes)
22. Data saturation	Was data saturation discussed?	Page 5 (Yes)
23. Transcripts returned	Were transcripts returned to participants for comment and/or correction?	No
**Domain 3: analysis and findings**		
Data analysis		
24. Number of data coders	How many data coders coded the data?	The first to fourth authors coded and compared results than all five authors reviewed together and confirmed the analysis.
25. Description of the coding tree	Did authors provide a description of the coding tree?	Page 5
26. Derivation of themes	Were themes identified in advance or derived from the data?	Page 5 (Themes were derived from the interview data)
27. Software	What software, if applicable, was used to manage the data?	No software was used. Analysis was done manually.
28. Participant checking	Did participants provide feedback on the findings?	No
Reporting		
29. Quotations presented	Were participant quotations presented to illustrate the themes/findings? Was each quotation identified? E.g., participant number	Quotations that support categories best were presented in table 2 and identified each expert number.
30. Data and findings consistent	Was there consistency between the data presented and the findings?	Yes
31. Clarity of major themes	Were major themes clearly presented in the findings?	Yes: Page 7~9, Table 2
32. Clarity of minor themes	Is there a description of diverse cases or discussion of minor themes?	Yes: Page 7~9, Table 2
